# Supplementary material for: TICTAC: target illumination clinical trial analytics with cheminformatics
Source: Front Bioinform. 2025 Jun 9;5:1579865. doi: 10.3389/fbinf.2025.1579865 (PMC12183303; doi:10.3389/fbinf.2025.1579865)
Supplement: Supplementary file 1 [file DataSheet1.pdf]

TICTAC Datasets Descriptions 02/19/2025

All files are accessible for download at: https://unmtid-dbs.net/download/TICTAC/

Filename	Description	Key Columns	Identifiers
aact_study_refs_trans_corrected.tsv.gz	References linked to clinical trials.	nct_id, pmid, year, reference_type	nct_id, pmid
disease_target_association_with_doid.tsv.gz	Disease-target-drug associations.	doid_uniprot, gene_symbol, nDiseases, nDrug, nStud, nPub, nct_ids, pmids	doid_uniprot, gene_symbol
ranking_info.tsv.gz	Detailed ranking information for numerical metrics.	doid_uniprot, meanRank, percentile_meanRank, meanRankScore, nDiseases_rank, nDrug_rank, nStud_rank, nPub_rank, nStudyNewness_rank, nPublicationWeighted_rank	doid_uniprot
filtered_DOID_1324.tsv.gz	Filtered disease-related associations (DOID:1324 - Liver Cancer).	doid_uniprot, nDiseases, nDrug, nStud, nPub, disease_term, drug_name, gene_symbol	doid_uniprot, gene_symbol
1324_provenance.tsv.gz	Associated provenance for liver cancer.	doid_uniprot, provenance_details	doid_uniprot

Filename	Description	Key Columns	Identifiers
filtered_DOID_9352.tsv.gz	Filtered disease-related associations (DOID:9352 - Diabetes Mellitus/Insulin Resistance).	doid_uniprot, nDiseases, nDrug, nStud, nPub, disease_term, drug_name, gene_symbol	doid_uniprot, gene_symbol
9352_provenance.tsv.gz	Associated provenance for diabetes mellitus/insulin resistance.	doid_uniprot, provenance_details	
